# Acute muscle swelling effects of a knee rehabilitation exercise performed with and without blood flow restriction

**DOI:** 10.1371/journal.pone.0278540

**Published:** 2022-12-22

**Authors:** Christopher J. Cleary, Trent J. Herda, Austin M. Quick, Ashley A. Herda

**Affiliations:** 1 Department of Health, Sport, and Exercise Sciences, University of Kansas Edwards Campus, Overland Park, Kansas, United States of America; 2 Department of Health, Sport, and Exercise Sciences, University of Kansas Lawrence Campus, Lawrence, Kansas, United States of America; Universita degli Studi di Milano, ITALY

## Abstract

This study assessed the acute effect of adding blood flow restriction (BFR) to quad sets on muscle-cross sectional area (mCSA), muscle thickness (MT), echo intensity (EI), and subcutaneous fat-normalized EI (EI_NORM_) of the superficial quadriceps muscles. Twelve males and 12 females (mean±SD; age (yrs): 21.4±2.9; stature (m): 1.76±0.1; body mass (kg): 77.7±2.9) performed 70 repetitions (one set of 30, three sets of 15 repetitions) of bodyweight quad sets separately on each leg, with or without BFR (CON) applied. Rating of perceived exertion was recorded following each set. Panoramic ultrasound images of the vastus lateralis (VL), vastus medialis (VM), and rectus femoris (RF) were captured prior to (PRE), immediately after (IMM-POST), 30- (30-POST), and 60-minutes after (60-POST) after exercise. Sex x condition x time repeated measures ANOVAs assessed differences at p<0.05 for each muscle and dependent variable separately. Although males had larger VM and VL mCSA and VL MT (p<0.05), there were no acute changes from PRE to IMM-POST (p>0.05). There was a 3-way interaction in VL mCSA (p = 0.025) which indicated BFR was greater than CON at IMM-POST by 7.6% (p = 0.019) for males only. Females had greater EI in the VM and VL than males (p<0.05), yet males had greater EI_NORM_ for each muscle (p>0.05) and EI_NORM_ did not change over time or treatment (p>0.05). The lack of changes in MT, EI, and EI_NORM_ indicate that unloaded quad sets do not provide a stimulus to promote fluid shifts or acute changes in muscle size with the exception of IMM-POST in the VL for males. Future research should attempt to elucidate the acute muscular responses of BFR application for lightly loaded rehabilitation exercises in the clinical populations for which they are prescribed.

## Introduction

Blood flow restricted (BFR) resistance training has become a widely used exercise training modality. BFR has become popular in general training for hypertrophy, training older adults, and in clinical practice following injury [1]. BFR resistance training is performed with significantly lower external loads than traditional high-load training and has been thoroughly applied in clinical and aging populations [2–4]. Although the exact physiological mechanisms of BFR resistance training remain unknown, it could be due to multiple systemic and/or local factors. These include, but are not limited to, swelling [5–7], metabolic stress [4], endocrine responses [8], and/or satellite cell recruitment [9].

Multiple studies have assessed the acute effect of BFR resistance exercise using low-load concentric movements on lower-limb muscle cross-sectional area (mCSA), muscle thickness (MT) [10–12] and echo intensity (EI), which has been suggested to represent changes in intracellular fluid [13]. The changes in intracellular fluid may be due to an increase in blood flow to the working musculature, cellular swelling, and/or metabolic buildup during and following activity [14]. These studies have presented mixed results, with Hackney et al. [11] showing no significant differences compared to baseline in rectus femoris mCSA immediately after and 5-minutes after BFR knee extensions when compared to low-load non-BFR exercises. Conversely, the MT of the vastus lateralis can be elevated immediately after BFR knee extensions with a concomitant decrease in EI [10] and MT can be elevated up to 3-hours after BFR knee extensions compared to baseline [12], purportedly due to local swelling. Additionally, the perceived effort of BFR-mediated “work” has been reported to be much higher than non-BFR movements, hence why a standard protocol for BFR includes only 20–30% 1-repetition maximum load during dynamic, concentric resistance exercises [11, 15].

Despite the ever-increasing amount of BFR research, there is a lack of literature on the addition of BFR to bodyweight only rehabilitation exercises. For example, following knee surgery, straight-leg raises, heel slides, and isometric quadriceps contractions “quad sets” are commonly prescribed [16–18], but without BFR. Typically, these are utilized in the early stages of rehabilitation (1–14 days post-surgery), with or without electrical stimulation or other therapeutic modalities [16]. If exercise modalities that maintain muscle mass after surgery can be utilized earlier in the recovery process, then perhaps the long-term deficits between a surgical limb and a non-surgical limb can be attenuated [19, 20]. If BFR-mediated resistance exercise can stimulate muscular changes in healthy adults [10, 12], studies should then assess the acute responses (e.g., changes in muscle size) of knee rehabilitation exercises performed with BFR, to determine if this provides a unique response. A substantial amount of the existing BFR literature has included both male and female participants, yet do not elucidate any sex-specific differences in BFR-driven muscular responses [19, 21, 22] and suggest future research should investigative if there are unique sex-related responses to BFR. To date, two investigations have compared sex and reported varying outcomes. Young et al. demonstrated similar trends between sex in vastus lateralis MT changes after BFR knee extensions [12] and Bell et al. also noted no differences in MT between sex after BFR resistance exercise [10].

Based on the potential efficacy of the addition of BFR to a knee rehabilitation exercise, the primary aim of this study was to assess acute changes in mCSA, MT, EI, and perceived exertion in males and females following quad sets with or without BFR. A secondary purpose was to assess any differences in these responses by sex. It was hypothesized that quad sets with BFR will require greater effort and elicit a muscle swelling response, as measured via mCSA, EI, and MT. It was also hypothesized that no differences would exist between males and females’ responses to the BFR or CON treatment applied.

## Materials and methods

**Fig 1:**
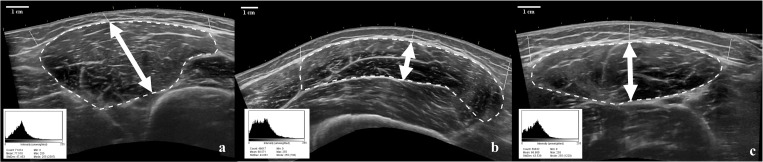
Image analysis parameters for the VM (a), VL (b), and RF (c). The arrows represent MT, straight lines represent subcutaneous fat thickness, and the dotted line represents mCSA. The histogram displays the uncorrected EI (mean) of each image.

A within-subjects repeated measures design was completed where participants performed unilateral quad sets separately on both legs with one leg exercising with BFR and the contralateral leg exercising without BFR (CON), under random assignment to the dominant and non-dominant legs. Condition order was randomized per participant with 10-minute rest between conditions. Panoramic ultrasound scans of the vastus lateralis (VL), vastus medialis (VM), and rectus femoris (RF) were captured prior to (PRE), immediately after (IMM-POST), 30-minutes (30-POST), and 60-minutes (60-POST) following the respective exercise protocols (S1 Fig 1 in S1 File). Rating of perceived exertion (RPE) was measured after each exercise set on a CR-10 scale [23]. Both conditions required participants to perform a total of 70 repetitions per leg. All participants provided written informed consent and completed a health and exercise history questionnaire prior to participation. All protocols were approved by the University IRB (#00147840) in accordance with the Declaration of Helsinki.

### Procedures

#### Participants

Twenty-four participants [12 males (age: 21.4±2.7 years; stature: 1.81±0.5 meters; body mass: 85.3±6.4 kg; and BMI: 26.1±2.1 kg∙m^2^) and 12 females (age: 21.3±3.1 years; stature: 1.70±0.7 meters; body mass: 70.2±8.6 kg; and BMI: 24.3±3.4 kg∙m^2^)] volunteered to participate in the present study from the university and local community. This sample size was determined from an a priori power analysis conducted in G*Power (G*Power 3.1, Kiel, Germany) using a moderate effect size of f = 0.25 and type I error rate of 0.05. A total of 24 participants was required to achieve statistical power of 0.80. The participants met the following inclusion criteria: male or female between 18–30 years, a body mass index between 18–30 kg∙m^-2^, and resistance training experience of at least 2x/week for the past 6 months, which indicated they were not sedentary, had experience with exercise, and is in agreement with previous samples [11, 12]. Participants were excluded if they had a history of cardiovascular, metabolic, or nervous system disorders, had any current orthopedic or musculoskeletal injuries, or recently (≤ 6-months) underwent lower-body surgery.

#### Ultrasound image acquisition

After the participant’s height and weight were taken and recorded, they laid supine on the examination table quietly for ~5 minutes [24] while the principal investigator explained the exercise techniques, prepared for the ultrasound measurements, and demonstrated the exercise technique. Ultrasound images of the VM, VL, and RF were captured at PRE, IMM-POST, 30-POST, and 60-POST of each condition (Fig 1). Participants remained supine on the examination table throughout the duration of the study visit, including between image acquisition timepoints. At PRE, the ultrasound measurements were taken consecutively, beginning with the leg that was randomized to exercise first. Panoramic images were captured in the transverse plane for the VL at 50% of the distance between the greater trochanter and the lateral epicondyle of the femur and for the RF at 50% of the distance from the superior border of the patella and the greater trochanter [25]. The VM was imaged on the medial aspect of the thigh, 10 cm above the superior pole of the patella [26]. A trained investigator captured all images using a Logiq e R7 diagnostic ultrasound (GE Healthcare, Wauwatosa, WI, USA) set in LOGIQ View^(R)^ to capture panoramic images with a linear array transducer (Model L4-12t-RS, 4.2–13.0 MHz; 12.7 x 47.1 mm probe surface area). Participants laid supine on a padded examination table with a 6” diameter PVC pipe placed under the popliteal fossa to elevate the thighs during ultrasound measurements. Scan depth was set to 6 cm for all participants, while gain and transducer frequency were 68 dB and 10 MHz, respectively, and these settings remained constant for each image acquisition. Water-soluble gel was applied to the skin and transducer to enhance image quality during all ultrasound measurements.

#### Exercise protocols

**Fig 2:**
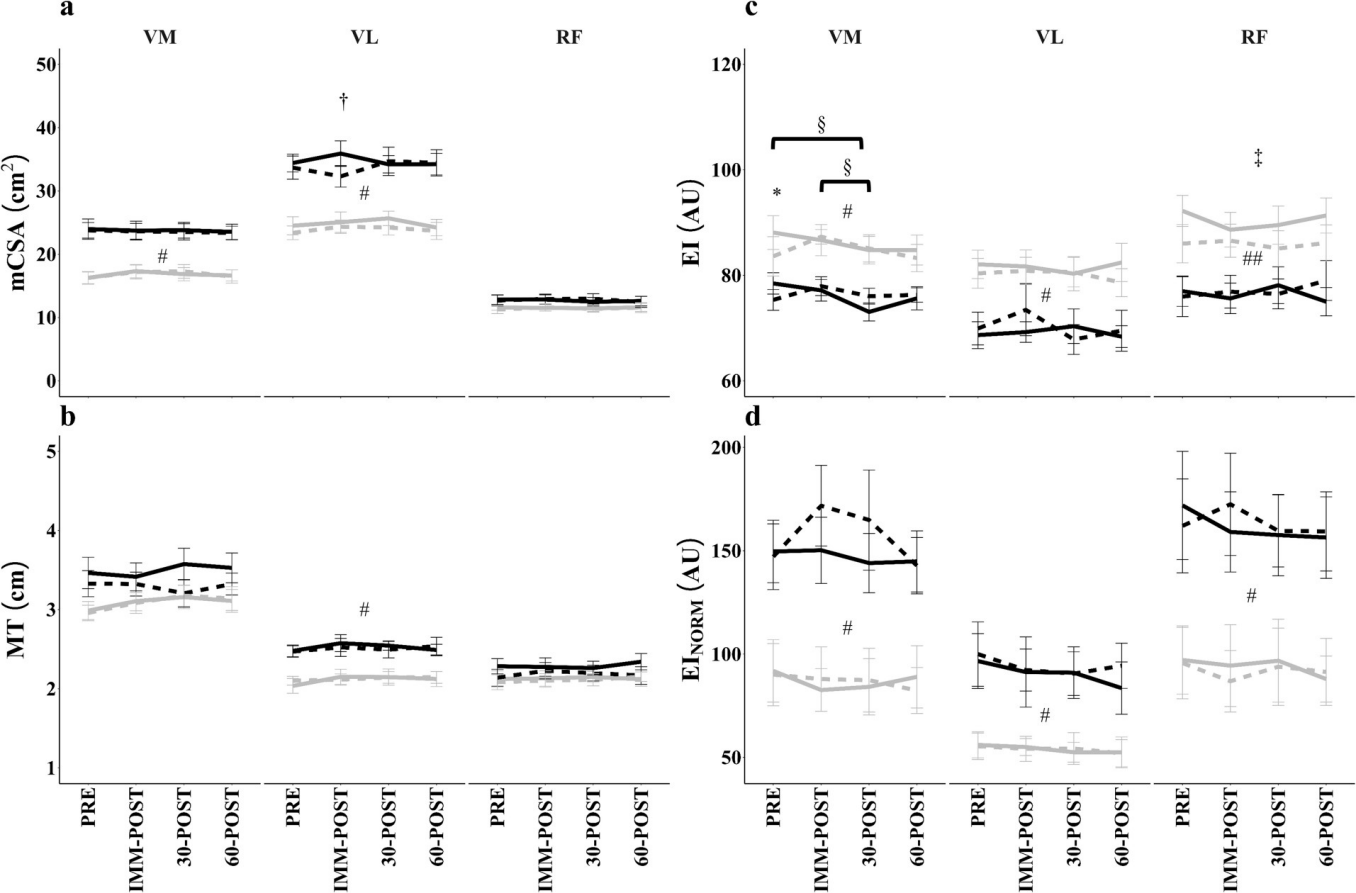
Changes in muscle cross-sectional area (a), muscle thickness (b), echo intensity (c), and subcutaneous fat-normalized echo intensity (d). Data represent mean±SE (males: black lines; females: grey lines; BFR: solid line; CON: dashed line). SE: Standard error. ^#^significant difference between sex collapsed across condition and time; ^##^significant differences between sex for BFR only; *significant differences between conditions at indicated timepoint collapsed across sex; ^†^significant difference between conditions for males at indicated timepoint; ‡significant difference between conditions for females only; §significant difference between timepoints.

Exercise condition order and leg allocation was randomized using Microsoft Excel’s (Microsoft Office, Microsoft, Redmond, WA, USA) randomization function (S1 Fig 1 in S1 File). After the PRE ultrasound scans, participants were provided demonstrations on proper quad sets technique: the exercising leg was extended while the non-exercising leg was bent at approximately 90° with the foot flat on the patient table and the participant’s hands placed on the table to support the trunk in an upright position (S1 Fig 2 in S1 File). Participants were cued to “squeeze the muscles in the front of your thigh, which may cause your knee to press down to the table” during all repetitions [17, 27]. One set of 30 repetitions followed by three sets of 15 repetitions with 30 seconds of rest between sets were performed separately on each leg [3, 19]. Ten minutes after the completion of the first exercise protocol on the first leg, the second exercise protocol (BFR or CON) was repeated on the opposite leg using the same procedures.

During the BFR condition, a 10-cm wide cuff (Smart Cuffs, Smart Tools Plus, Strongsville, OH, USA) was applied to the proximal portion of the participant’s thigh, near the inguinal crease, and inflated to 80% of the participants arterial occlusion pressure as determined by an internal doppler sensor. The cuff remained inflated between sets and was removed upon conclusion of the final set. Total occlusion time including inflation was recorded. The CON condition was performed on the contralateral limb without BFR applied. After each set, RPE on a CR-10 scale was measured [23]. Based on the randomized nature of limb and condition set-up, twelve participants completed the CON condition first with the other 12 completing the BFR condition first and ten participants exercised with the left leg first. Three participants reported the left leg as their dominant leg through being asked which leg they would prefer to kick a ball with [28].

### Ultrasound image analysis

All images were saved on the ultrasound hard drive and subsequently exported to an external USB drive for analyses in ImageJ (NIH, Bethesda, MD, USA). Prior to image analyses, each image was blinded by an external investigator, so that the image analyst would not be influenced by the timepoints or conditions [12]. Similar to previous research [29], MT (cm) was defined as the distance between the superior and inferior borders of the muscle at measured at 50% of the horizontal distance of the muscle belly, mCSA (cm^2^) was determined by manually tracing the entirety of the muscle’s border, and EI was determined as mean gray-scale value (0–255 arbitrary units: AU) from the mCSA region of interest. Subcutaneous fat thickness (cm) was measured at three locations: the distance from the superficial aponeurosis to the skin at 50% of the midpoint of the muscle belly, and the lateral and medial ends of the muscle belly. To account for differences in subcutaneous fat between participants and sexes, EI was normalized to subcutaneous fat by dividing EI by the average subcutaneous fat thickness of the three locations as indicated on Fig 1. Our laboratory has previously reported ultrasound image analysis reliability from intra-class correlation coefficients ranging from 0.98–0.99 for quadriceps femoris in ImageJ [29].

### Statistical analyses

All data are reported as mean±SD, unless otherwise noted. All data are available in the supplemental file (S2 File). Three separate three-way 2x2x4 [sex (male vs. female) x condition (BFR vs. CON) x time (PRE vs. IMM-POST vs. 30-POST vs. 60-POST)] repeated measures analyses of variance (ANOVA) were conducted to assess differences in mCSA, MT, EI, and normalized EI for each muscle (VM, VL, and RF). A separate three-way [sex x condition x time (set 1 vs. set 2 vs. set 3 vs. set 4)] ANOVA assessed changes in RPE. If a significant interaction was identified, follow-up analyses of additional 2-way or 1-way ANOVAs, t-tests and post-hoc analyses with Bonferroni corrections were conducted. Additionally, an independent t-test was conducted to any descriptive characteristic differences between the sexes. Partial eta square (η_p_^2^) effect sizes were generated and interpreted as trivial (<0.01), small (0.01–0.06), moderate (0.06–0.14), or large (>0.14) [30] effects. All analyses were performed in SPSS v. 27 (IBM, Armonk, NY, USA). Data were considered significant with p<0.05.

## Results

### Characteristic differences between males and females

Males were significantly taller (mean difference = 0.10 m; percent difference = 6.1%; p = 0.001) and heavier (mean difference = 15.1 kg; percent difference = 21.5%; p<0.001) than the female participants, yet similar in age (p = 0.95) and body mass index (p = 0.117). There was no significant difference between sexes for BFR pressure (males: 183.0±16.2 mmHg; females: 175.7±17.2 mmHg; p = 0.30). The average total occlusion time including inflation and exercise was 9.07±0.97 min with no difference between sexes (p = 0.681).

### Muscle cross-sectional area

There was no 3-way interaction in the VM (p = 0.85, η_p_^2^ = 0.012) or the RF (p = 0.73, η_p_^2^ = 0.019) (Fig 2A). There were also no 2-way (condition x time, sex x condition, or time x sex) interactions in the VM or the RF. Furthermore, there were no main effects for condition or time in either the RF or VM (all p>0.05). There was, however, a main effect for sex (p<0.001, η_p_^2^ = 0.481) as males (23.66±4.11 cm^2^) had larger VM mCSA than females (16.78±3.29 cm^2^). Yet RF mCSA was similar (p = 0.184, η_p_^2^ = 0.079) between sexes (males: 12.74 ± 2.49 cm^2^; females: 11.50 ± 1.81 cm^2^).

In the VL, there was a 3-way interaction for mCSA (p = 0.025, η_p_^2^ = 0.131). When the full model was decomposed and split by sex, there was neither a 2-way (condition x time) interaction (p = 0.811, η_p_^2^ = 0.028) nor was there a main effect for time or condition (p = 0.075–0.253) in the females. For males, there was a 2-way (condition x time) interaction (p = 0.008, η_p_^2^ = 0.298), yet the decomposed interaction revealed no changes across time in either condition (BFR: p = 0.358, η_p_^2^ = 0.092; CON: p = 0.056, η_p_^2^ = 0.202). At IMM-POST, BFR was significantly greater than CON by 3.60±4.54 cm^2^ (p = 0.019). All other timepoints were similar between conditions (p = 0.442–0.871). Additionally, when the 3-way interaction was split by condition, the BFR condition indicated no 2-way (sex x time) interaction (p = 0.384, η_p_^2^ = 0.045), but there was a main effect for sex (p<0.001, η_p_^2^ = 0.527) as males (34.68±5.2 cm^2^) had larger VL mCSA than females (24.86±4.48 cm^2^). In CON, there also was a 2-way (sex x time) interaction for VL mCSA (p = 0.047, η_p_^2^ = 0.113). Follow-up analyses revealed no changes across time for males (p = 0.056, η_p_^2^ = 0.20) or females (p = 0.358, η_p_^2^ = 0.092). However, collapsed across time for CON, males (33.77±6.54 cm^2^) had larger (p<0.001) VL mCSAs than females (23.90±3.83 cm^2^). There were no sex x condition interactions at PRE (p = 0.760, η_p_^2^ = 0.004), IMM-POST (p = 0.103, η_p_^2^ = 0.116), 30-POST (p = 0.242, η_p_^2^ = 0.062), and 60-POST (p = 0.655, η_p_^2^ = 0.009). Collapsed across condition, there was a main effect for sex at all timepoints as males had larger VL mCSAs than females (mean difference±SD; all p<0.001, PRE = 10.1±6.84 cm^2^; IMM-POST = 9.40±2.14 cm^2^; 30-POST = 9.49±7.03 cm^2^; 60-POST = 10.39±7.47 cm^2^).

### Muscle thickness

For MT (Fig 2B), there were no significant 3-way interactions (VM: p = 0.207, η_p_^2^ = 0.066; VL: p = 0.300, η_p_^2^ = 0.013; RF: p = 0.132, η_p_^2^ = 0.083), nor were there any significant 2-way interactions (VM range: p = 0.14–0.49; VL range: p = 0.41–0.86; RF range: p = 0.22–0.95). There was a main effect for sex in the VL (p = 0.001, η_p_^2^ = 0.384) as males (2.51±0.27 cm) had larger MT than females (2.12±0.25 cm). Males and females had similar MT in the VM (males: 3.39±0.53 cm; females: 3.09±0.42 cm; p = 0.130, η_p_^2^ = 0.101) and RF (males: 2.24±0.33 cm; females: 2.12±0.26 cm; p = 0.353, η_p_^2^ = 0.039). There were no additional main effects for condition (VM: p = 0.138, η_p_^2^ = 0.097; VL: p = 0.956, η_p_^2^<0.001; RF: p = 0.089, η_p_^2^ = 0.126) nor time (VM: p = 0.185, η_p_^2^ = 0.070; VL: p = 0.069, η_p_^2^ = 0.101; RF: p = 0.642, η_p_^2^ = 0.025).

### Echo intensity

For EI (Fig 2C), there were no 3-way interactions (VM: p = 0.36, η_p_^2^ = 0.047; VL: p = 0.84, η_p_^2^ = 0.012; RF: p = 0.88, η_p_^2^ = 0.010). However, there was a condition x time interaction in the VM (p = 0.049, η_p_^2^ = 0.11) when collapsed across sex. Also, there was a main effect for sex in the VM (p = 0.036, η_p_^2^ = 0.18) as females (83.8±12.6 AU) had greater EI than males (75.7±6.9 AU) collapsed across time and condition. The decomposed condition x time model indicated no change across time for CON (p = 0.79, η_p_^2^ = 0.015). But there was a main effect for time for BFR (p = 0.002, η_p_^2^ = 0.19) as 30-POST (77.37±11.2 AU) was less than both PRE (83.28±10.3 AU, p<0.001) and IMM-POST (81.9±9.9 AU, p = 0.016). Further, EI in the VM during BFR at PRE was significantly greater than CON at PRE (mean difference = 4.8±7.4 AU, p = 0.006) with no other differences between timepoints for conditions (p-range = 0.18–0.28). There were no additional 2-way interactions (sex x condition: p = 0.051, η_p_^2^ = 0.162 and time x sex: p = 0.396, η_p_^2^ = 0.050) in the VM.

There were no 2-way interactions in the VL (sex x condition: p = 0.86, η_p_^2^ = 0.001; time x sex: p = 0.74, η_p_^2^ = 0.019, condition x time: p = 0.64, η_p_^2^ = 0.025) nor main effects for condition (p = 0.24, η_p_^2^ = 0.063) or time (p = 0.15, η_p_^2^ = 0.078). There was a main effect for sex (p<0.001, η_p_^2^ = 0.44) as females (80.4±10.2 AU) had greater VL EI than males (64.6±9.7 AU).

Lastly, there was a sex x condition interaction in the RF (p = 0.014, η_p_^2^ = 0.25) but no other 2-way interactions (time x sex: p = 0.67, η_p_^2^ = 0.023, condition x time: p = 0.77, η_p_^2^ = 0.017) nor was there a main effect for time (p = 0.45, η_p_^2^ = 0.039). Decomposition of the sex x condition interaction in the RF indicated that females had greater EI during BFR (mean difference = 16.3±4.3 AU, p<0.001) but not CON (mean difference = 9.41±4.6 AU, p = 0.054). For the male participants, there were no differences between conditions (p = 0.35). While for the females, RF EI was greater in BFR (90.4±10.5 AU) than CON (85.4±11.2 AU, p = 0.016).

For EI_NORM_ (Fig 2D), there were no 3-way interactions (VM: p = 0.59, η_p_^2^ = 0.03; VL: p = 0.74, η_p_^2^ = 0.18; RF: p = 0.33, η_p_^2^ = 0.02) nor any 2-way interactions (VM range: p = 0.051–0.23, VL range: p = 0.62–0.85, RF range: p = 0.49–0.67). There were also no main effects for condition (VM: p = 0.23, η_p_^2^ = 0.07; VL: p = 0.63, η_p_^2^ = 0.01; RF: p = 0.99, η_p_^2^<0.001) nor time (VM: p = 0.20, η_p_^2^ = 0.07; VL: p = 0.27, η_p_^2^ = 0.06; RF: p = 0.27, η_p_^2^ = 0.06). Yet there was a main effect for sex in each muscle (VM: p = 0.005, η_p_^2^ = 0.31; VL: p = 0.006, η_p_^2^ = 0.29; RF: p = 0.015, η_p_^2^ = 0.24) as males had greater EI_NORM_ (VM: 151.9±57.6 AU; VL: 92.4±43.6 AU, RF: 162.3±71.4 AU) than females (VM: 86.9±46.9 AU; VL: 53.9±20.9 AU; RF: 92.9±56.9 AU).

### Rating of perceived exertion

**Fig 3:**
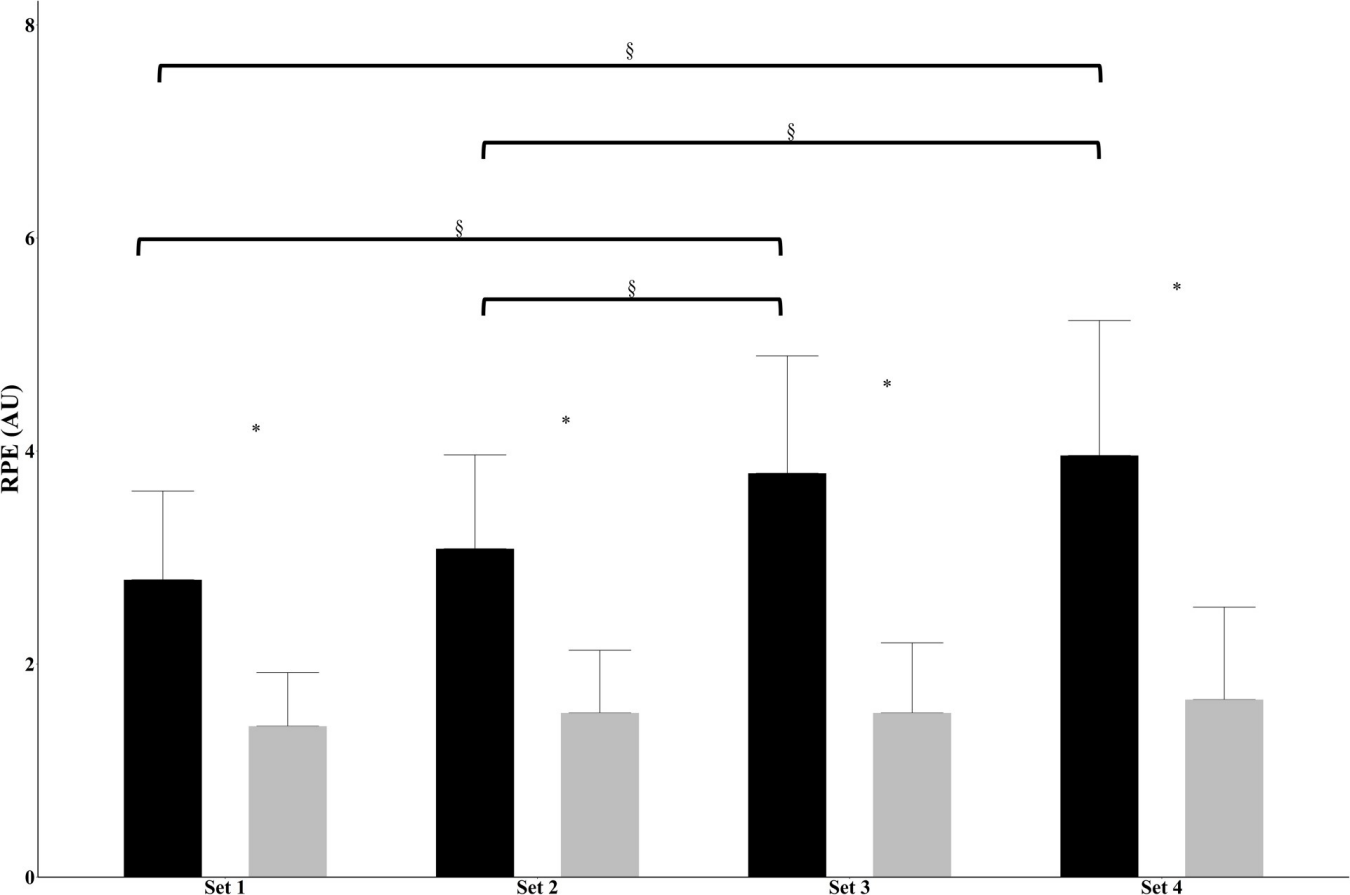
Muscle rating of perceived exertion between conditions and time. Data represent mean±SD and collapsed across sex (BFR: black bars, CON: grey bars). SD: Standard deviation. *significant differences between conditions at indicated timepoint, collapsed across sex; §significant difference between timepoints.

There was no 3-way interaction (sex x condition x time) (p = 0.075, η_p_^2^ = 0.099), and no condition x sex (p = 0.622, η_p_^2^ = 0.011), or time x sex (p = 0.783, η_p_^2^ = 0.016) interactions. However, there was a 2-way interaction for condition x time (p<0.001, η_p_^2^ = 0.499), collapsed by sex (Fig 3). Subsequent analyses determined RPE was similar over time for CON (p = 0.144, η_p_^2^ = 0.075). In BFR there was a main effect for time (p<0.001, η_p_^2^ = 0.579), as set 3 (3.79±1.10 AU) was significantly greater than set 1 (2.79±0.83 AU, p<0.001) and set 2 (3.08±0.88 AU, p<0.001). Set 4 (3.96±1.27 AU) was significantly greater than sets 1 (p<0.001) and 2 (p<0.001). Sets 1 and 2 (p = 0.097) and sets 3 and 4 (p = 0.970) were similar.

## Discussion

The purposes of this study were to assess changes in mCSA, MT, and EI of the three superficial quadriceps femoris muscles after a knee rehabilitation exercise performed with and without BFR between males and females. The main finding of this study was that there were no significant changes in muscle size (mCSA or MT) from PRE values in either condition or sex despite greater RPE in the latter sets with BFR. However, BFR induced an approximately 7.6% difference compared to CON in VL mCSA for males at IMM-POST. Additionally, EI was influenced at some time points following the BFR treatment for both sexes and may represent a fluid shift 30 minutes after exercise in the VM. Females had greater EI than males in the VM and VL. However, there were no changes in EI_NORM_, although males had greater EI_NORM_ than females collapsed across condition and time. Independent of sex, all participants reported greater exertion in the latter sets of the BFR condition.

Despite the recent rise in the amount of studies utilizing BFR within a rehabilitation setting [19, 20, 31], there remains a lack of evidence that demonstrates the acute muscular responses to specific rehabilitation exercises performed with BFR. The current study’s results presented a temporary increase in mCSA at IMM-POST in males which may have been caused by the BFR quad sets, but this was a modest 4.0% and insufficient to be significantly greater than any other time point. These results contrast with previous studies that have identified significant changes in muscle size variables (MT and mCSA) of 10.7–12.6% from PRE to after exercise following knee extensions performed with BFR [10–12]. However, the exercises used included concentric, low-load (20–30%) voluntary contractions at varying inflation pressures (140 mmHg) or relative percentages [50% arterial occlusion pressure (AOC)]. BFR has been traditionally performed with low-loads ranging from 20–30% of one-repetition maximum (1-RM) [10, 12]. It is possible the quad sets exercise performed against bodyweight with 80% AOC or without BFR was an insufficient stressor to acutely change muscle size, suggesting load may be required to elicit change. The present results indicated there was no significant change in MT for any muscle following the quad sets exercise with or without BFR. Similarly, Park et al. [32] reported no change in MT of the VL 0, 5, or 10 minutes after unloaded passive knee extensions performed with and without BFR applied. Despite the differences in body position (supine v. seated) and exercise (quad sets v. passive knee extensions), the results of the present study align with the conclusion of Park et al. [32], in that a stimulus of no load BFR is insufficient to result in muscle swelling in healthy participants. Expected differences existed between males and females VL MT and mCSA at all time points. The differences in quadriceps femoris muscle size between males and females has been previously documented for VL mCSA [33]. The lack of differences between sexes in the RF mCSA has been also reported by Myers et al. [34].

In the present study, the change in EI was only evident in the VM which may indicate the level of muscular involvement during a quad set contraction is greater in the VM than the other quadriceps muscles [35]. This concept is supported by the work of Gryzlo et al., as the VM and VL had greater electromyographic amplitudes than the RF during a shortened knee extension moving from 15 degrees of knee flexion to 0 degrees of knee flexion, similar to a quad set [36]. It is interesting to note that there was no change in EI from PRE to IMM-POST in the BFR condition, rather, the difference was indicated at 30-POST only in the VM. BFR can result in greater hemodynamic responses versus low-load non-BFR exercise [11, 15]. Previous methodology suggests resting supine prior to musculoskeletal area measurements as body position could influence fluid shifts [37], however, the predominant literature have indicated no effect of rest time on EI [24, 38, 39]. This may suggest that the change in the VM as presented in the present study may have been driven by some other physiological mechanism such as the intervention and not due any positional shift. Similarly, acute changes in EI have been postulated to be representative of fluid movement within the region [10, 13], as an acute stimulus cannot induce immediate structural changes. Collectively, females have been shown to have greater EI of the quadriceps femoris when compared to males [30]. In the present study, there were no changes in EI_NORM_ across time or condition. This is interesting as BFR has been reported to result in greater hemodynamic responses versus low-load non-BFR exercise [11, 15], suggesting BFR could reduce EI values following exercise. Following low-load exercise an increase in EI of the biceps brachii was shown by Hill et al. with and without BFR [40] and in the VL and RF after non-occluded knee extensions by Muddle et al. [41], while Wong et al. noted inconsistent changes in the biceps brachii after BFR exercise [42]. Due to the differences in methodologies between these and other studies and the inconsistent findings of the present study to go along with those presented by previous research, there does not appear to be a consensus on what happens to EI after BFR exercise [43]. Males had significantly greater EI_NORM_ than females in each muscle by ~70%, which opposes when EI is reported without subcutaneous fat consideration [33]. This could be due to males in general having lower EI than females and less subcutaneous fat on the anterior thigh. These shifts may be influenced by factors such as exercise type (isometric versus dynamic constant external resistance) and may differ between skeletal muscles. Future research should continue to investigate the response of EI to BFR exercise and attempt to elucidate what it represents, physiologically. Lastly, it is important to note that the differences EI observed in the present compared to previous research could be due to factors such as subcutaneous fat normalization and differences in ultrasound equipment and settings [29, 33].

The participants reported BFR to result in higher perceived exertion than CON by 1.86 AU (~120%) across all sets, which is in line with pervious literature [11, 15]. Specifically, Hackney et al. reported an RPE increase of ~6.0 AU from baseline to the peak response during BFR knee extensions [11]. Poton and Polito reported the highest RPE from low-intensity BFR resistance exercise over high- or low-intensity resistance exercise without BFR [15]. Potentially, the pressure of the occlusion cuff alone could have resulted in greater discomfort and exertion than the CON condition, although a 10-cm cuff width has been reported to be the least painful [3, 22]. Despite the increase in the RPE in the latter sets that included BFR in the present study, the lack of muscular swelling (MT and mCSA) suggests the unloaded contractions may not have been a sufficient stimulus, even if the participants perceived them to be more difficult.

Limitations of this study include that the participants did not have physical or functional deficits, such as injury or atrophy, to elicit the intended response to the unloaded quad sets. Further, the differences in ultrasound equipment and may limit our ability to compare EI findings across studies. Additionally, the physiologic meaning of changes to EI have been interpreted in vastly different scenarios and settings, which warrants further evaluation and perhaps higher-level validity (i.e., comparison to fat or fluid shifts as evaluated by magnetic resonance imaging of near-infrared spectroscopy), which the present study did not pursue. To best elucidate the potential of BFR in clinical populations, future directions of similar investigations may need to include the manipulation of resistance training related variables (e.g., load, repetitions, tempo) with BFR in an injured and rehabilitating population. Modified loaded rehabilitation exercises should be explored, such as single leg raises with ankle weights. Future efforts should also continue to investigate the differences between sexes in response to BFR exercise, especially as females have been overall greatly underrepresented in the exercise physiology literature.

## Conclusion

Overall, the present study provides insight to the muscular response of males and females completing isometric quadriceps contractions, or quad sets, with and without BFR. Perceived exertion was greater towards the end of exercise sessions, although there was largely a lack of effect from BFR quad sets on significant changes across time in mCSA and MT of the VL and VM. No acute fluid shifts were indicated as there were no changes in EI, normalized to subcutaneous fat, in any of the muscles in either condition. Some acute fluid shifts were indicated as the corrected EI of the VM changed following BFR, yet males and females responded similarly under both conditions. The RF was not influenced in any way for either sex or condition. As quad sets have been traditionally utilized only in clinical populations following lower extremity surgeries, it is possible that clinical populations may have differential responses than the ones observed in the present study, which warrants future research.

## Supporting information

S1 File Timeline of study procedures (S1 Fig 1) and exercise set-up with blood flow restriction cuff applied (S1 Fig 1). (PDF)

S2 File All available data of the study in csv format. (CSV)
