# Supplementary material for: Acute muscle swelling effects of a knee rehabilitation exercise performed with and without blood flow restriction
Source: PLoS One. 2022 Dec 22;17(12):e0278540. doi: 10.1371/journal.pone.0278540 (PMC9778495; doi:10.1371/journal.pone.0278540)

S1 Fig 1. Timeline of study procedures.

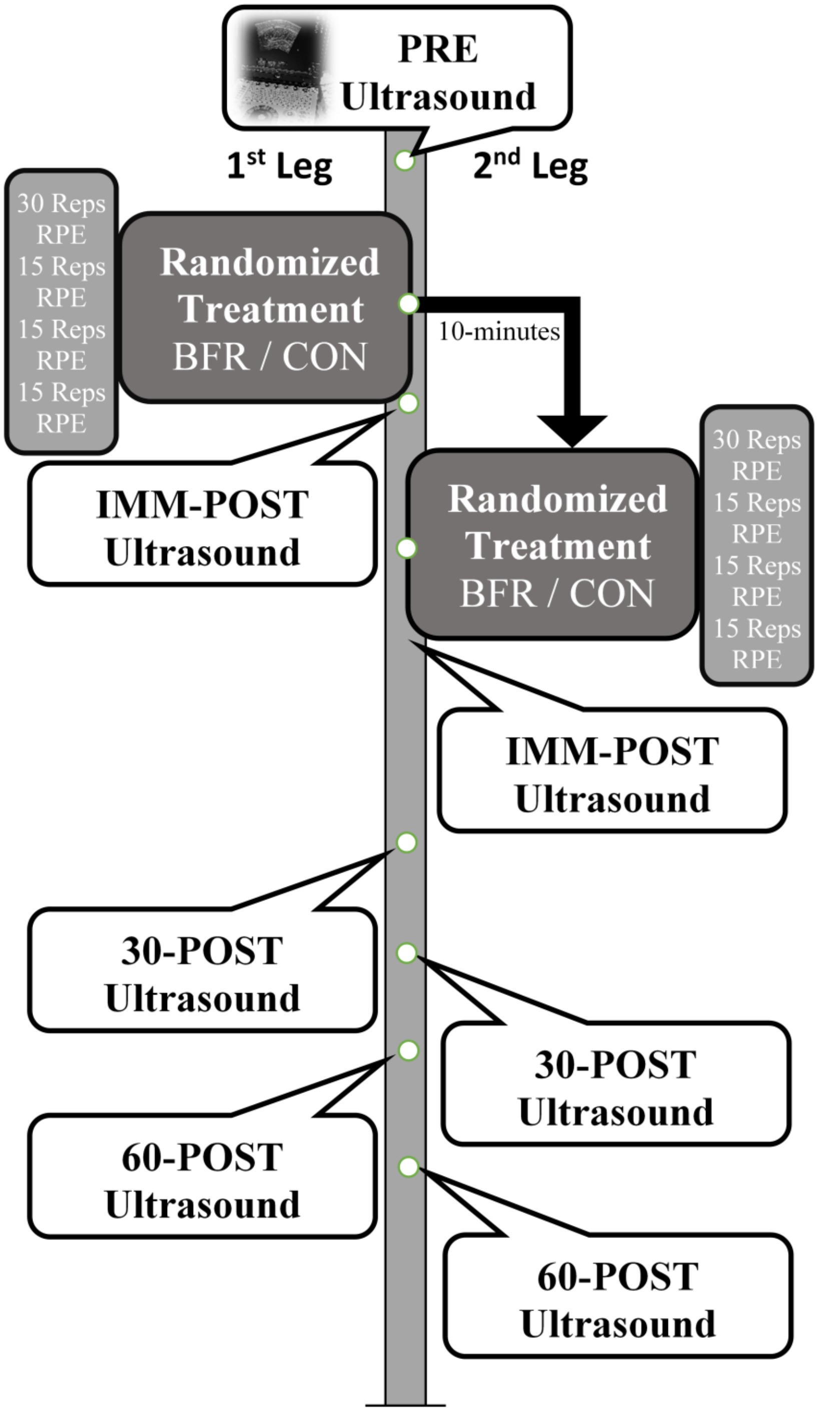

S1 Fig 2. Exercise set-up with blood flow restriction cuff applied.

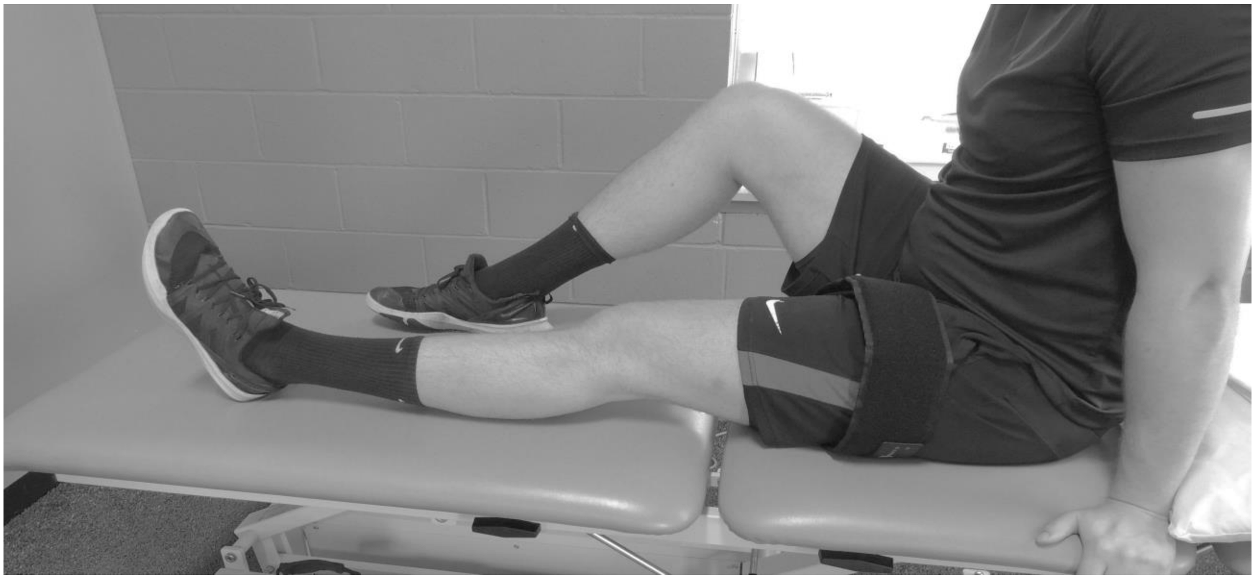

Supplement: S1 File — (PDF) [file pone.0278540.s001.pdf]
